# Immune-related hepatic adverse events in renal cell carcinoma patients treated with immune checkpoint inhibitors: a retrospective study

**DOI:** 10.1038/s44276-025-00178-7

**Published:** 2025-09-16

**Authors:** Amer Saleh, Viktor Grünwald, Thomas Hilser, Christopher Darr

**Affiliations:** University Hospital Essen, Essen, Germany

## Abstract

**Background:**

Immune checkpoint inhibitors (ICIs) have revolutionized the treatment of advanced renal cell carcinoma (RCC), but their use is associated with immune-related adverse events, including hepatic adverse events (irHAEs).

**Methods:**

We retrospectively analysed 105 RCC patients treated with ICIs as first-line therapy between 2018 and 2023 at the University Hospital of Essen. Patients were categorized by the development of irHAE, defined per CTCAE grading v5.0. Multivariable logistic regression was used to identify risk factors, while Kaplan-Meier survival analyses evaluated PFS and OS.

**Results:**

Among the cohort, 16.19% (*n* = 17) developed irHAE, while 8.57% (*n* = 9) experienced higher-grade events. Combination therapy with tyrosine kinase inhibitors (TKIs) was associated with a higher likelihood of irHAE (OR: 7.69, *p* = 0.037) compared to ICI-only regimens, with cabozantinib showing a significantly shorter time to onset (35 vs. 84 days; *p* < 0.001). Patients with a BMI ≥ 25 had a significantly increased risk (*p* = 0.011). Differences in PFS (18.63 vs. 19.87 months; *p* = 0.099) and OS (27.80 vs. 23.87 months; *p* = 0.36) were not statistically significant.

**Conclusions:**

The combination of ICI with TKI posed higher risks for irHAE in RCC patients. While survival outcomes were unaffected, the results underscore the need for tailored monitoring and management. Prospective studies are warranted to refine therapeutic approaches.

## Background

Renal cell carcinoma (RCC), known for its poor responsiveness to conventional chemotherapy, accounts for 80–85% of kidney cancers and represents a significant global health burden [1, 2]. A hallmark of RCC is the intricate interplay between tumour biology and the immune system, driven by complex immunogenetic factors. The immune microenvironment of RCC tumours is enriched with immune cells, including tumour-infiltrating lymphocytes, macrophages, and regulatory T-cells, which contribute to both, tumour progression and immune evasion. Furthermore, RCC tumours frequently overexpress immune checkpoint molecules, such as PD-L1, that suppress T-cell activity and promote tumour survival [3].

Systemic therapies have revolutionized the treatment landscape for metastatic RCC. Targeted therapies, such as tyrosine kinase inhibitors (TKI; e.g., sunitinib, pazopanib) and mTOR inhibitors (e.g., everolimus), disrupt key pathways in tumour growth and progression. More recently, immune checkpoint inhibitors (ICIs) have emerged as transformative agents, reactivating T-cells to recognize and attack cancer. ICIs have significantly improved survival outcomes and are now a cornerstone of treatment for advanced metastatic RCC, especially in combination regimens [4]. However, their use is accompanied by challenges, as the heightened immune activation they induce can lead to a distinct spectrum of immune-related adverse events (irAEs) that set them apart from conventional therapies [5]. Among these, immune-related hepatic adverse events (irHAEs) are particularly concerning due to their potential severity and implications for treatment continuity [6].

The interplay between ICIs, the immune system, and the liver underscores the need for a deeper understanding of irHAEs. While clinical trials have yielded valuable data on the safety and efficacy of ICIs, they often involve carefully selected patient populations that may not reflect the diversity of real-world RCC patients with comorbidities [7]. Consequently, real-world evidence on the incidence, risk factors, and management of irHAEs across different therapeutic regimens in RCC patients remains limited.

This retrospective study aims to address these gaps by evaluating the incidence, risk factors, and clinical outcomes of irHAEs in RCC patients treated with ICIs in a monocentric patient cohort. By identifying key risk factors and assessing the impact of irHAEs on treatment outcomes, this study seeks to provide insights for further research regarding the use of ICI for RCC patients.

## Methods

### Study design and population

We conducted a retrospective cohort study to evaluate the incidence, risk factors, and outcomes of irHAEs in adult RCC patients treated with ICI. The study was performed at the University Hospital of Duisburg-Essen, with ethical approval number 23-11568-BO obtained from the institutional review board. Patient data were anonymized to ensure confidentiality. Eligible patients were adults with histologically confirmed, locally advanced or metastatic RCC who received ICI-based therapy as first-line treatment between 2018 and 2023. Inclusion criteria required complete medical records with baseline laboratory data, imaging reports, and follow-up assessments. Patients without follow-up or those with incomplete records were excluded. irHAEs were defined based on the Common Terminology Criteria for Adverse Events (CTCAE) grading V5.0, with grade 1 being the mildest and higher grades being associated with more elevated values of liver function tests. The primary endpoint was the incidence of irHAEs. Secondary endpoints included progression-free survival (PFS), overall survival (OS).

### Statistical analysis

Descriptive statistics were used to summarize patient demographics, tumour and treatment patterns. Continuous variables were analysed using the Student’s t-test or Mann-Whitney U test, and categorical variables were assessed with chi-squared or Fisher’s exact tests. Risk factors for irHAEs were identified using logistic regression models, and Kaplan-Meier curves with log-rank tests were employed to compare PFS and OS between groups. Cox proportional hazards models were used to evaluate the impact of irHAEs on survival, adjusting for potential confounders. Statistical significance was defined as *p* < 0.05. All analyses were conducted using SPSS (version 28). Kaplan-Meier curves were produced using RStudio (version 4.3).

## Results

**Fig. 1:**
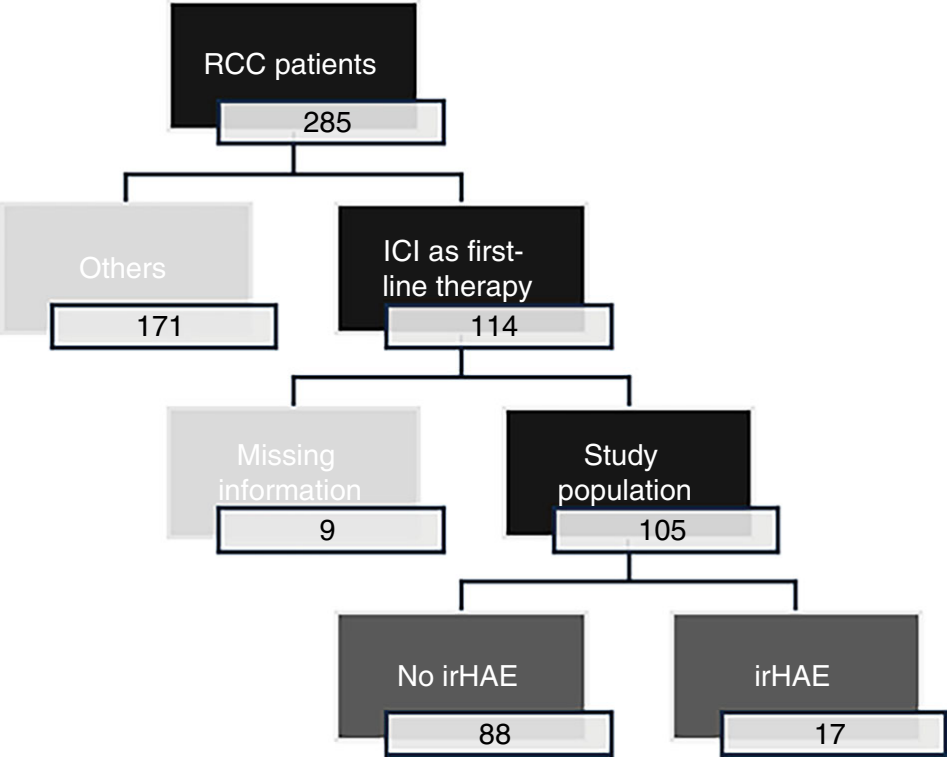
Flowchart of patient selection based on inclusion and exclusion criteria. RCC renal cell carcinoma, ICI immune checkpoint inhibitors, irHAE immune-related hepatic adverse events.

As per the available records, there were 285 patients treated for RCC, 114 patients received ICI as first line therapy. From these, 9 patients were excluded for missing relevant information regarding their RCC diagnosis, therapy or follow-up. As a result, our study included 105 patients. Among those, 7 patients (6.66%) had a preexisting liver condition (hepatitis, steatosis hepatis, etc). irHAEs were observed in 17 patients (16.19%), with mild irHAE (grade I-II) in 8 patients (7.62%) and severe irHAE (grade III and IV) in 9 patients (8.57%) Fig. 1.

### Demographics

Both subgroups based on irHAE status showed similar distribution of age (62.52 years vs. 63.36 years, *p* = 0.651) and gender (*p* = 0.887). Patients with irHAEs were more likely to be overweight or obese compared to those without (76.5% vs. 67%, *p* = 0.035, OR 34,42). Most patients (85.81%) had between 0–5 comorbidities, with no significant differences in comorbidity distribution between those with and without irHAEs (*p* = 0.822). ECOG and IMDC scores were comparable between groups (*p* = 0.094 and 0.48, respectively).

### Tumour and treatment characteristics

Clear cell RCC (ccRCC) was the most common subtype, comprising 73.33% of cases. Among patients with irHAEs, ccRCC was observed in 88.24%, compared to 70.45% in those without irHAEs (*p* = 0.835). The majority of patients (72.38%) had Stage IV disease at initial diagnosis. Stage distribution did not significantly differ between those with and without irHAEs (*p* = 0.365). Metastases were most commonly observed in the lungs, bones, and liver. Adrenal metastases were significantly associated with irHAEs (29.41% vs. 9.68%, *p* = 0.011, OR 23,06).

**Fig. 2:**
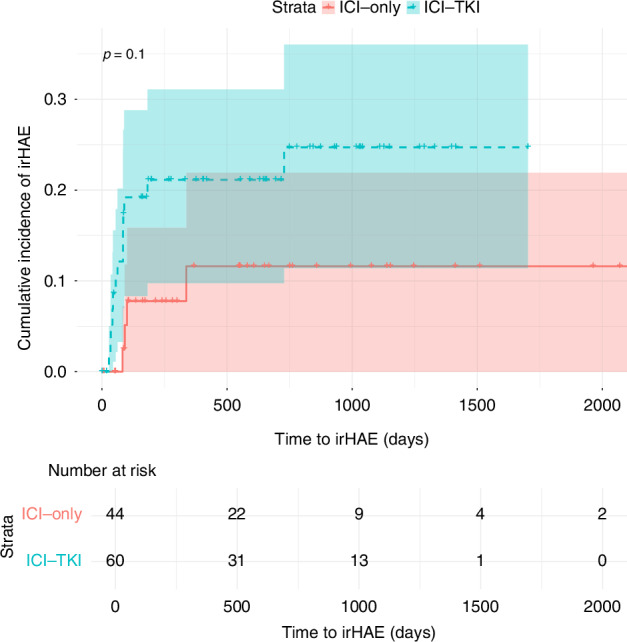
Kaplan-Meier curve for time to onset of irHAE by therapy group. Solid line: ICI-only group, dashed line: ICI-TKI.

**Fig. 3:**
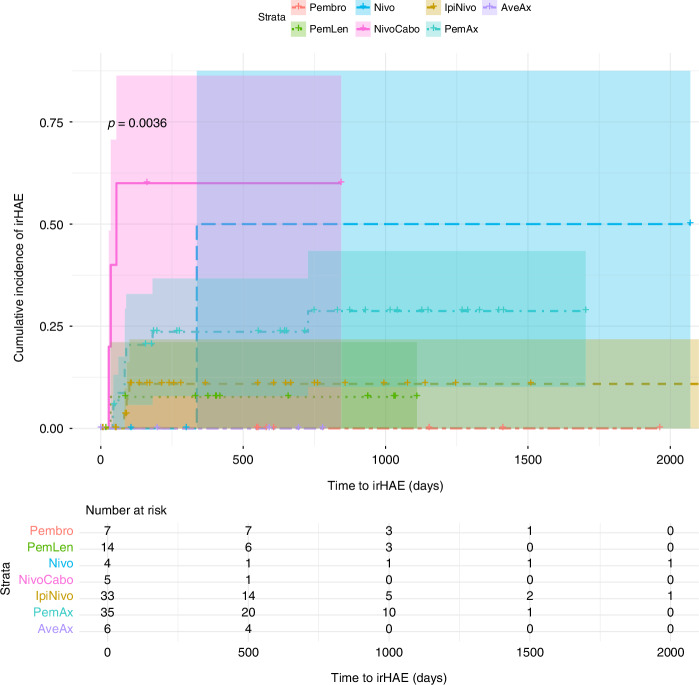
Kaplan-Meier curve for time to onset of irHAE by therapy regimen. Solid line: nivolumab with cabozantinib, long dashed line: nivolumab alone, dashed dotted line for pembrolizumab with axitinib, short dashed line: ipilimumab with nivolumab, dotted line: pembrolizumab with lenvatinib. Other lines overlapping.

**Fig. 4:**
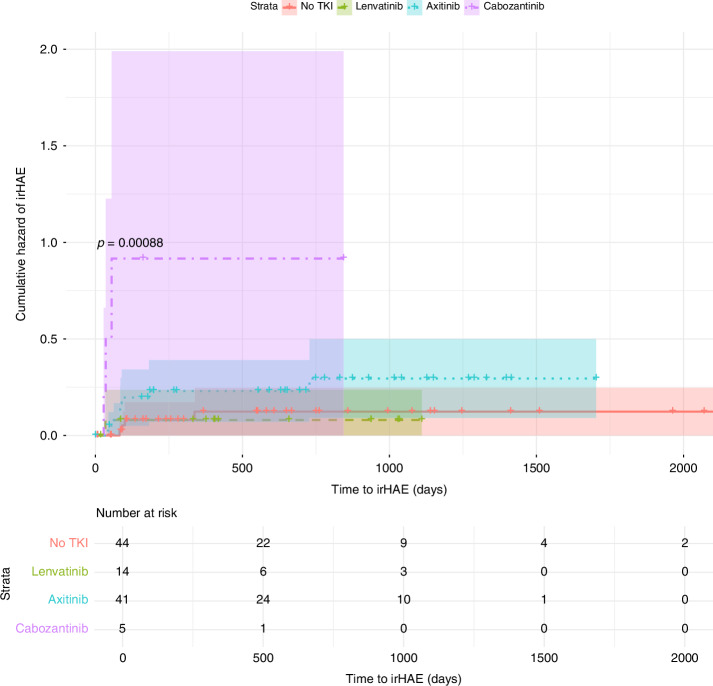
Kaplan-Meier curve for time to onset by TKI agent. Solid line: no TKI, dashed line: lenvatinib, dotted line: axitinib, dashed-dotted line: cabozantinib.

Combination therapies involving ICIs and TKIs were more common among patients who developed irHAEs compared to those on ICI without TKI (76.47% vs. 23.53%, *p* = 0.037, OR 7,69) Tables 1–4. Although there was a trend towards higher severity of irHAEs in the ICI-TKI group, the difference was not statistically significant. Among patients with lower-severity irHAEs, 5 (62.50%) had received ICI-TKI therapy, compared to 8 (88.88%) in the higher-severity group. Among the ICI- TKI group, combination with cabozantinib was associated with the highest risk of irHAEs, with a median time to onset of 35 days (IQR 13.00) compared to 84 days (IQR 4.00) for other ICI-only and TKI-ICI regimens (*p* < 0.001). Pembrolizumab was the most frequently used agent (53.3%), followed by nivolumab (40.95%), and ipilimumab (31.42%). No significant differences in irHAE incidence were observed based on the specific ICI used Figs. 2–4.

**Table 1:** Patients’ characteristics and frequency of irHAE.

Parameter	No irHAE (*n* = 88)	With irHAE (*n* = 17)	*p*-value
Age at time of therapy – Mean (SD)	63,36 (12.76)	62.52 (9.88)	0.651
BMI – Mean (SD)	27.70 (6.04)	28.39 (4.18)	0.296
Gender (M) – n (%)	63 (71.59%)	12 (70.58%)	0.887
Overweight – n (%)	59 (67.04%)	13 (76.47%)	0.035
Smoking history – n (%)	17 (19.32%)	4 (23.53%)	0.794
Diabetes mellitus – n (%)	11 (12.50%)	2 (11.76%)	0.197
Hypertension – n (%)	49 (55.68%)	7 (41.18%)	0.205
Preexisting hepatic conditions – n (%)	5 (5.68%)	2 (11.76%)	0.165
Number of comorbidities – n (%)			0.822
•1–2	37 (42.05%)	7 (41.18%)	
•3–5	39 (44.32%)	8 (47.06%)	
•6+	12 (13.64%)	2 (11.76%)	
ECOG – n (%)			0.094
•0	58 (65.91%)	9 (52.94%)	
•1	22 (25.00%)	6 (35.29%)	
•2	3 (3.41%)	1 (5.88%)	
•3–4	5 (5.69%)	1 (5.88%)	
Histology – n (%)			0.835
•ccRCC	62 (70.45%)	15 (88.24%)	
•pRCC	11 (12.50%)	1 (5.88%)	
•sRCC	5 (5.68%)	1 (5.88%)	
•undifferentiated	7 (7.95%)	0 (0.00%)	
•Other	3 (3.41%)	0 (0.00%)	
Stage at time of initial diagnosis – n (%)			0.365
•1	9 (10.23%)	1 (5.88%)	
•2	3 (3.41%)	1 (5.88%)	
•3	12 (13.64%)	0 (0.00%)	
•4	61 (69.32%)	15 (88.24%)	
Location of Metastases – n (%)			
•Lung	52 (55.91%)	9 (52.94%)	0.169
•Bone	30 (32.26%)	8 (47.06%)	0.828
•Liver	14 (15.05%)	2 (11.76%)	0.394
•Lymph nodes	22 (23.66%)	2 (11.76%)	0.805
•Adrenal glands	9 (9.68%)	5 (29.41%)	0.011
Time to Therapy – Mean (SD)	38 (76.97)	17.84 (26.16)	0.185
Therapy group – n (%)			
•ICI-only	39 (44.32%)	4 (23.53%)	0.037
•ICI-TKI	49 (55.68%)	13 (76.47%)

**Table 2:** irHAE Patients’ characteristics and irHAE grade.

Parameter	Grade I–II (*N* = 8)	Grade III–IV (*N* = 9)	*p* value
Age at time of therapy – Median (IQR)	63 (5)	64 (13)	0.451
BMI – Median (IQR)	30.59 (3.39)	25.20 (2.06)	0.075
Gender – n (%)			
- Males	6 (75%)	6 (66.66%)	0.306
ICI-only vs. ICI-TKI – n (%)			0.494
- ICI-only	3 (37.50%)	1 (11.11%)	
- ICI-TKI	5 (62.50%)	8 (88.88%)

**Table 3:** Patients’ characteristics and irHAE hazard.

	Significance	Hazard ratio
**BMI**	0,535	0,95
**Obesity**	0,157	4,54
**Gender**	0,853	0,87
**ECOG**	0,141	1,78
**Number of Comorbidities**	0,661	0,91
**Smoking history**	0,519	0,61
**Hypertension**	0,392	1,75
**Diabetes mellitus**	0,628	0,62
**Other Liver Conditions**	0,224	0,32
**Histology**	0,999	
**Stage**	0,240	1,58
**Bone metastasis**	0,920	0,94
**Lung metastasis**	0,240	2,16
**Liver metastasis**	0,863	1,17
**Adrenal metastasis**	0,002	9,61
**Lymph metastasis**	0,618	1,59
**Age at therapy**	0,805	0,99
**Time to therapy**	0,693	0,10
**Therapy Group (ICI-TKI)**	0,035	4,55

**Table 4:** irHAE incidence by therapy regimen.

Therapy group	Therapy regimen (*n*)	Incidence *n* (%)
ICI	Nivolumab [4]	1 (25.00%)
	Ipilimumab with nivolumab [33]	3 (9.09%)
	Pembrolizumab [7]	0
ICI-TKI	Pembrolizumab with axitinib [35]	9 (25.71%)
	Pembrolizumab with lenvatinib [14]	1 (7.14%)
	Nivolumab with cabozantinib [6]	3 (50.00%)
	Avelumab with axitinib [6]	0

#### Management strategy

Due to irHAE, therapy doses were modified in 41.18% (*n* = 7) of irHAE cases, while treatment was interrupted in 76.47% (*n* = 13) and discontinued in 41.18% (*n* = 7) of cases. Steroids were used in 76.47% (*n* = 13) patients. Excluding outliers, the mean duration of steroid therapy was 7.40 weeks (SD 6.85 weeks), While higher-grade irHAE required longer therapy durations, the difference was not statistically significant (*p* = 0.524). Kaplan-Meier analysis showed a shorter median time to response in the ICI-only group (30 days) compared to the ICI-TKI group (56 days). However, the difference was not statistically significant (*p* = 0.15). A Kaplan-Meier analysis across TKI agents revealed axitinib had the longest median response time (80 days) compared to cabozantinib (42 days) and lenvatinib (57 days). The differences were not statistically significant (*p* = 0.18).

#### Clinical outcome

**Fig. 5:**
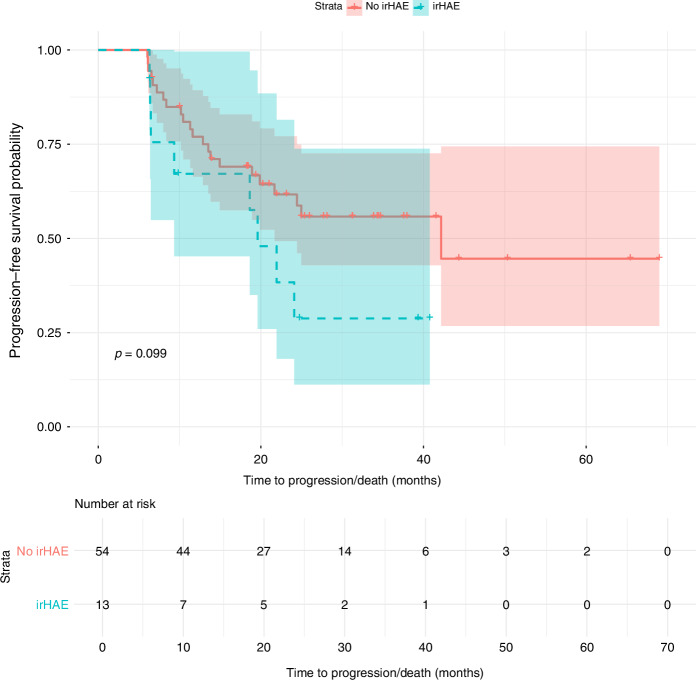
Kaplan-Meier curve for PFS by irHAE status. Solid line: patients with no irHAE, dashed line: patients with irHAE.

**Fig. 6:**
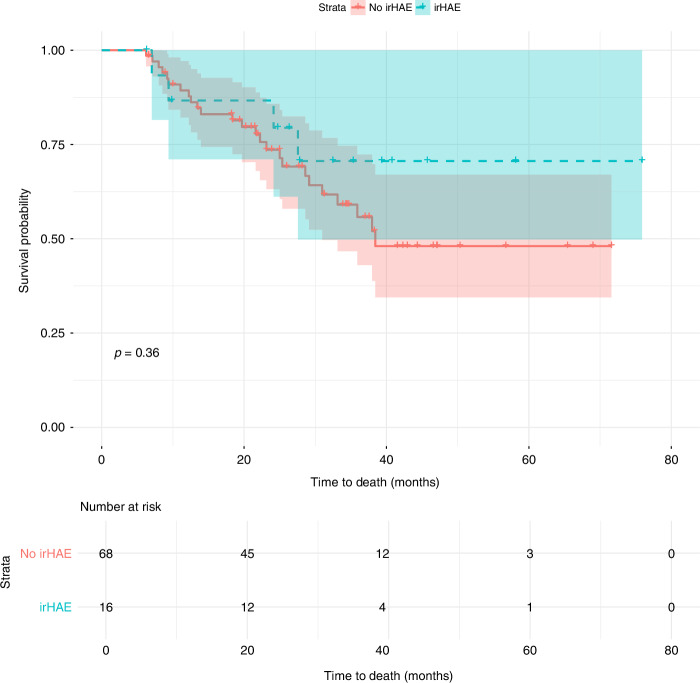
Kaplan-Meier curve for OS based on irHAE status. Solid line: patients with no irHAE, dashed line: patients with irHAE.

PFS was not significantly different between patients with and without irHAEs (18.63 vs. 19.87 months; *p* = 0.099). OS was also similar between groups (27.80 vs. 23.87 months; *p* = 0.36) Figs. 5–6.

## Discussion

### Incidence of irHAE

The incidence of irHAE in RCC patients treated with ICI, with and without TKI treatments, varies across studies. While hepatic involvement is less common among irAEs, it remains clinically significant due to its potential severity, including risks of terminal liver dysfunction and impact on the continuation of therapy. Pooled analyses of clinical trials across cancer types report overall hepatotoxicity rates of 1–15% for all grades and 1–10% for grade 3–4 events [8]. Specific to RCC, reported incidences are largely consistent, influenced by factors such as the type of ICI, combination therapy, and baseline liver function [9]. In our study of 105 RCC patients receiving ICIs, 17 patients (16.19%) developed irHAEs, aligning with previous studies. The slightly higher rates of severe irHAEs than mild irHAE in our study could reflect differences in baseline patient characteristics, the inclusion of a broader range of therapeutic regimens, or real-world treatment dynamics that differ from controlled clinical trial settings.

### Demographics and Risk of irHAE

Aging impacts immune function through immunosenescence, altering both innate and adaptive immunity. This may theoretically influence irAE risk in ICI therapy. However, evidence remains inconsistent; some studies report stable irAE rates across age groups, while others suggest increased risk in older patients due to comorbidities, cancer types, or specific ICIs [10, 11]. In our cohort, no significant age-related differences in irHAE incidence or severity were observed, supporting the notion that age alone is not a decisive factor.

Furthermore, sex hormones, such as oestrogens and androgens, seems to possibly modulate immune checkpoint protein function and ICI responses [12]. Meta-analyses indicate that women may be more prone to specific irAEs, such as thyroid dysfunction, while other irAEs show no clear gender trends [13]. Moreover, the type of ICI appears to play a role: women seem more affected by CTLA-4 inhibitor-related irAEs, whereas men show higher risks with PD-1/PD-L1 inhibitors [14]. Though limited data suggest higher irHAE rates in women, our study found no statistically significant differences, only a trend toward increased rates in female patients [15].

Beyond age and sex, comorbidities further complicate irAE risk, as conditions like autoimmune diseases, allergies, chronic kidney and lung diseases putatively alter immune responses [14]. While some studies have linked higher comorbidity scores (e.g., Charlson or NCI Comorbidity Index) to increased irAE risk and poorer outcomes [16, 17], others have found no significant association [18, 19]. Specific data on RCC patients and irAEs, including irHAEs, remain sparse. A retrospective analysis of 4,270 patients indicated that conditions such as diabetes and chronic kidney disease influence treatment outcomes and adverse event profiles, often delaying the onset of irAEs and complicating their differentiation from comorbidity-related effects [20].

Pre-existing liver conditions, historically excluded from clinical trials, like hepatitis B, hepatitis C, and cirrhosis may increase irHAE risk due to compromised hepatic function. Evidence is however conflicting in this regard, with some studies suggesting increased hepatotoxicity risk in these patients, and others reporting no significant differences [21–24]. Taking into account an age-standardized prevalence of liver cirrhosis and other liver diseases of approximately 833 cases per 100,000 people in Germany [25], patients with previous liver conditions were rare in our cohort (6.66%). Only two of seven patients with chronic liver diseases developed irHAEs with no statistically significant difference.

Metabolic disorders, particularly obesity and diabetes, play a complex role in irHAE risk, exhibiting distinct but interconnected effects on immune regulation and treatment outcomes. Obesity is characterized by chronic low-grade inflammation and dysregulation of immune function, driven by metabolic overload and the active role of adipose tissue as an immunological organ [26]. Despite being a risk factor for multiple cancers, obesity paradoxically enhances the efficacy of ICI therapies, potentially due to higher PD-1 expression on exhausted CD8 + T cells, making them more responsive to PD-1 inhibitors [27]. Some studies suggested, on the other hand that overweight and obese patients might be more likely to develop irAEs compared to those with normal BMI [28, 29]. In our study cohort, BMI itself was not found to be a risk factor for irHAEs. Being obese or overweight, however, aligning with the available literature, was a significant predictor with an odds ratio of 34.42, indicating a strong increase in the likelihood of irHAE in overweight or obese patients. Though not statistically significant in our study, we also observed that BMI might predict irHAE grade, with a higher BMI being associated with a lower probability of experiencing severe irHAE grades. Similar findings were reported by McQuade et al., who analysed 3772 patients with advanced cancer. Their study found that obesity was associated with an increased incidence of any-grade irAEs in patients treated with nivolumab monotherapy. However, the incidence of grade 3 or 4 irAEs (severe irAEs) was not significantly higher in obese patients compared to those with normal BMI [30].

### Tumour characteristics

While meta-analyses examining the relationship between RCC histology and irAE risk are scarce, some evidence suggests ccRCC may carry a higher irAE risk due to its immunogenic properties, driven by VHL gene alterations and VEGF signalling. Conversely, non-clear cell RCC subtypes, such as papillary and chromophobe RCC, exhibit lower immunogenicity and reduced responsiveness to ICIs [31, 32]. Our study found no significant association between RCC histological subtypes and the incidence of irHAEs. Clear cell RCC (ccRCC) was the predominant subtype in both irHAE and non-irHAE groups, with minimal variation in the distribution of non-clear cell subtypes. The limited representation of non-clear cell RCC subtypes restricts our ability to identify subtype-specific patterns in irAEs. Further research is needed to clarify these associations, particularly for non-clear cell RCCs.

As for the TNM classification, while larger tumours are associated with higher tumour burden and increased immune activation [33], our findings suggest that tumour size alone may not directly correlate with irHAE risk. This could reflect the complex interplay between the immune system and the tumour microenvironment, where factors such as immunogenicity, baseline immune status, and microenvironmental characteristics play a more prominent role in influencing immune activation and toxicity.

Regarding metastasis, one retrospective observational cohort study conducted on patients with NSCLC treated with ICIs noted that irAEs are influenced by the location of metastases. Specifically, the absence of bone metastases was associated with a higher likelihood of developing irAEs. This can however be otherwise attributed to the poor prognosis and eventually the shorter treatment duration of patients with bone metastases other than to a possible change in immune reactivity. The study found no associations were observed between other metastatic sites, like the brain, liver, or adrenal glands, and the occurrence of irAEs [34]. In our study, metastases were most commonly located in the lungs and bones, with similar distributions between the irHAE and non-irHAE groups. Lung metastases, which were the most frequent in our study, were not significantly associated with an increased risk of irHAE. Similarly, bone and liver metastases, which were also prevalent, did not show a significant relationship with irHAE.

Interestingly, adrenal metastasis was the only metastatic site associated with a statistically significant increased risk of irHAE. While no previous studies assessed the association between metastatic involvement of the adrenal glands and the risk of developing irAE, the GETUG-AFU-26 NIVOREN, provided insights into how adrenal metastasis in patients with metastatic RCC might affect the response to immunotherapy. Specifically, it was found that patients with adrenal metastases had poorer outcomes when treated with nivolumab. This subgroup showed lower PFS and OS compared to patients without adrenal metastases, suggesting a reduced efficacy of immunotherapy in these patients [35]. The underlying mechanism for this observation remains unclear and further analysis is needed to understand the factors contributing to this finding. Moreover, given the limited sample size in our study, additional research with larger cohorts is required to validate these results.

### Treatment characteristics

The time to treatment initiation (TTI) has been linked to survival outcomes in cancer patients, with delays often resulting in poorer prognoses [36]. While timely initiation of ICI therapy is critical in metastatic RCC, particularly within the first year after diagnosis as per IMDC criteria [37], its association with irAEs, including irHAE, has been minimally explored. A study on underrepresented lung cancer patients found no significant relationship between TTI and irAE incidence within 12 months of ICI initiation [38]. Similarly, our study observed no clear association between TTI and irHAE risk, suggesting that irHAEs are more likely driven by immune checkpoint inhibition mechanisms rather than therapy timing.

Our study identified that combining TKIs with ICI significantly increased the risk of irHAE, with a 7.69-fold higher likelihood compared to ICI mono- or dual therapy. This was as well evident in a Kaplan-Meier analysis that indicated significant differences in the cumulative hazard of irHAE across various treatment regimens, as evidenced by a Log-Rank test p-value of <0.01 for specific regiments.

TKIs such as cabozantinib, axitinib, and lenvatinib target critical pathways in angiogenesis, tumour growth, and metastasis, including VEGFR, MET, and AXL pathways. Despite their therapeutic benefits, these agents exhibit off-target effects, particularly hepatotoxicity. Mechanistically, TKIs impair mitochondrial function, reducing ATP production and increasing reactive oxygen species, which can lead to hepatocellular damage. They also inhibit the bile salt export pump (BSEP), causing intracellular bile acid accumulation and cholestatic liver injury. Furthermore, TKI metabolism in the liver produces reactive intermediates that can bind to cellular macromolecules, resulting in direct hepatocyte damage [39]. When combined with ICIs, these mechanisms may synergistically amplify the risk of hepatic injury.

Data from trials such as Keynote-427 reported a comparable safety profile for pembrolizumab monotherapy with previous studies. However, when combined with TKIs like lenvatinib, pembrolizumab has demonstrated increased toxicity [40, 41]. Similarly, the JAVELIN Renal 101 trial found significant toxicities associated with avelumab and axitinib combinations [42]. Notably, the phase III COSMIC-313 trial demonstrated a higher incidence of irHAE with cabozantinib, nivolumab, and ipilimumab triplet therapy compared to nivolumab and ipilimumab doublet therapy in untreated advanced RCC patients. ALT elevations occurred in 19% of patients receiving the triplet therapy versus 4% in the doublet group [43]. A meta-analysis of 43 randomized controlled trials further supported these findings, revealing that systemic therapies combining ICIs with other agents significantly increased hepatotoxicity risk. This included both events of any grade and severe cases, with an OR of 2.16 and 2.72 respectively [44]. These results corroborate our observation and underscore the necessity of vigilant monitoring and prompt intervention for patients receiving these combination regimens.

Among TKI agents, we found that cabozantinib was significantly associated with a higher risk of irHAE compared to other TKIs when combined with ICIs. Specifically, the combination of cabozantinib with nivolumab demonstrated a significant association with irHAE, suggesting a markedly elevated risk of hepatic toxicity relative to other TKI-ICI combinations. Moreover, cabozantinib not only increased the likelihood of irHAE but also led to their earlier onset compared to other TKIs. Cabozantinib is a multitargeted TKI that inhibits VEGFR, MET, and AXL pathways, which are vital for tumour progression and angiogenesis but also play critical roles in maintaining normal liver function. This mechanism likely underpins the significantly heightened risk of hepatotoxicity observed in cabozantinib-ICI regimens, particularly those involving nivolumab [45]. A meta-analysis examining hepatotoxicity across cancer therapies confirmed that cabozantinib is associated with an increased incidence of liver enzyme elevations across all grades, with relative risks surpassing those of other TKIs and non-TKI treatments. The elevated risk may be attributed to the broad molecular targeting of cabozantinib, extensive hepatic metabolism, and potential mitochondrial toxicity [46], findings that align with our observations.

### Clinical outcome

The relationship between irAEs and clinical outcomes in RCC patients receiving ICI has been extensively studied. Numerous reports indicate that the occurrence of irAEs, such as skin and endocrine toxicities, is associated with improved PFS and OS across various cancers, including RCC [47–49]. Moreover, the management of irAEs has generally been shown not to compromise the efficacy of ICI therapy [50]. However, the specific impact of irHAEs on clinical outcomes remains less well-defined. In our study, Kaplan-Meier analysis revealed that patients who developed irHAE experienced a more pronounced decline in progression-free probability over time compared to those without irHAE. Nonetheless, the log-rank test demonstrated no statistically significant difference between the two groups. This finding aligns with reports suggesting that while irAEs are broadly associated with favourable outcomes, the relationship between irHAEs and clinical outcomes may not adhere to this pattern uniformly [51]. Variability in the characteristics and prognostic implications of immunotherapy-related liver injury complicates its association with treatment efficacy.

Similarly, our analysis revealed that irHAE does not significantly influence OS. This is consistent with other studies, where irAEs, including irHAE, have not been shown to directly correlate with decreased survival, while some studies suggested that patients who develop irHAE tend to experience significantly improved OS compared to those who do not across all grades of hepatitis [52]. The high percentage of censored cases—with 61% overall and 76.5% of irHAE patients still alive at the end of the study—reflects that many patients had not reached the endpoint of death by the time of analysis. This limits the statistical power to detect differences in OS between those with and without irHAE.

Our results showed no significant difference in PFS and OS between patients with irHAE and those without. This observation underscores that the occurrence of irHAE does not necessarily imply poorer outcomes in terms of disease progression for RCC patients undergoing ICI therapy. Importantly, even in cases requiring treatment interruptions, dose modifications, or therapy discontinuation due to irHAE, these adjustments did not adversely impact the duration of disease control. This highlights the need for further research to better understand the nuanced role of irHAEs in influencing the therapeutic trajectory of RCC patients receiving ICI.

## Conclusion

This study provides an important contribution to the understanding of irHAE in RCC patients undergoing ICI therapy. The findings underscore that irHAE, while not common, is a significant clinical concern that leads often to interruption or discontinuation of therapy. Patients receiving combination therapy with TKIs and ICIs were at a particularly higher risk, and specific agents, such as cabozantinib, substantially increase the risk and accelerate the onset of irHAE. The study also demonstrated that obesity and the presence of adrenal metastases were associated with a higher risk of developing irHAE. Despite the clinical significance of irHAE, the study did not find statistically significant differences in PFS or OS between patients who developed irHAE and those who did not. This suggests that with appropriate and timely management strategies, it is possible to control irHAE without compromising the overall efficacy of ICI treatment.

These findings highlight the critical importance of individualized treatment planning, factoring in patient-specific risks, disease biology, and the distinct toxicological profiles of therapies. Further research is warranted to address the limitations of this study and deepen the understanding of irHAE. The retrospective nature of this study inherently limits its ability to establish causal relationships. This focus on a single institution constrains as well the applicability of the conclusions to settings with different patient populations or treatment approaches. The small sample size of the irHAE cohort also precluded the ability to study the effects of re-exposure to ICIs after the occurrence of irHAE, a crucial consideration for understanding long-term therapeutic strategies. Survival analysis was also limited by the small size. Prospective, multi-centre studies with larger and more diverse patient populations are needed to validate these findings and explore additional risk factors. Additionally, mechanistic studies should investigate the biological pathways underlying irHAE to identify potential biomarkers for early detection and therapeutic targets for prevention and treatment.

## Data Availability

Data is available on request to the corresponding author.
